# Cephalometric Investigation of First Cervical Vertebrae Morphology and Hyoid Position in Young Adults with Different Sagittal Skeletal Patterns

**DOI:** 10.1155/2014/159784

**Published:** 2014-07-24

**Authors:** Seher Gündüz Arslan, Neval Dildeş, Jalen Devecioglu Kama

**Affiliations:** ^1^Department of Orthodontics, Faculty of Dentistry, University of Dicle, 21280 Diyarbakır, Turkey; ^2^Private Practice, Elazığ, Turkey; ^3^Private Practice, Izmir, Turkey

## Abstract

The aim of this retrospective study was to examine hyoid bone position and C1 (atlas) morphology in males and females and analyze these parameters with respect to different sagittal skeletal patterns via cephalometry, with the goal of identifying cephalometric norms. Lateral cephalometric radiographs from 120 individuals (average age: 21.1 ± 2.9 years) were classified according to their ANB angle (Class I, II, or III) and used to assess 14 parameters. Class I and II patients showed significant differences in Hy-NSL, Hy-PD, Hy-CVT, Lum, and a-p measurements. These parameters were consistently larger in males than in females. Intergroup comparisons among males showed significant differences in the SNA, ANB, Hy-CVT, X, and Z measurements. The hyoid was positioned more inferiorly and anteriorly and was more prominent in males than in females in all groups. Among participants exhibiting a Class I skeletal pattern, C1 was also larger in the anterior-posterior direction in males than in females. In the sagittal plane, the hyoid was positioned similarly in males with either Class I or III skeletal patterns but was positioned posteriorly in males with a Class II skeletal pattern. In addition, the vertical position of C1 varied with sagittal skeletal pattern in males.

## 1. Introduction

The head and neck have a balanced relationship similar to a lever-and-pivot system, with its fulcrum at the level of the occipital condyle. To maintain an upright position, muscles exert equal but opposing anterior and posterior forces on the occipital condyles [1] such that the head remains balanced over the first cervical vertebra (C1), also known as the atlas. C1 is an irregular ring-shaped bone made up of two lateral masses joined at the front and back by the short anterior and long posterior arches [2]. Importantly, no intervertebral disk develops between the atlas and the second cervical vertebra (C2), the axis [3].

Because the atlas represents the transition between the skull and the axial skeleton, it is of particular interest in orthodontics [4], and it is thought that the dimensions of this vertebra affect aspects of both the facial skeleton and the cervical column [5]. For example, variation in the dimensions of C1, as well as head and neck posture, were associated with differences in craniofacial morphology involving the cranial base [4–8], the upper airway space [9], occlusion [4], and temporomandibular disorders [10, 11]. Treuenfels [12] observed that the inclination of the atlas is associated with sagittal jaw position in that the anterior arch of the atlas shows a more cranial position in progenic compared to orthogenic patients.

Changes in head posture [13, 14] and changes in the inclination of the mandible [15] also influence the position of the hyoid bone. The hyoid, which is positioned between the mandibular symphysis and the larynx in the front of the neck, joins together the tongue, mandible, cranial base, sternum, scapula, thyroid cartilage, and pharynx and is influenced by these structures [16]. The hyoid bone has no bony articulation with any other bone but is instead suspended in the soft tissue via ligaments and muscles [17]. The function of this structure is to maintain the positional balance of the respiratory passage as well as respiratory sufficiency by anchoring the tongue. It also has an important role in tongue function and in maintaining an upright head position and cranial balance [18]. Because of its unique structure, the position of the hyoid bone changes with head posture and body position and moves during various oral functions in close association with tongue activity [19, 20]. Without the hyoid, the position of the head could not be maintained with the same degree of fine control [21].

In the literature, it has been shown that changes in mandibular position are related to hyoid bone changes and that the hyoid position adapts to anterior-posterior changes in head posture [22–24]. Furthermore, studies on the relationship between the hyoid and the facial skeleton and cervical column have indicated that the hyoid-cervical relationship is more stable than the relationship of the hyoid to the skull and mandible [22, 25, 26]. Consequently, studies examining different malocclusions can reveal the role of the hyoid bone in the dentofacial system and the response of mandibular development to these malocclusions.

Modern orthodontics studies dentomaxillofacial structures, including the head and neck. When examining these structures and their relationships with one another, the position of hyoid bone with respect to other structures becomes important, especially for preventing relapse after orthodontic and orthognathic surgical treatment. Accordingly, the aim of this retrospective study was to examine hyoid bone position and C1 (atlas) morphology in males and females and analyze these parameters with respect to different sagittal skeletal patterns via cephalometry, with the goal of identifying cephalometric norms.

## 2. Methods

A total of 120 patients (60 males, 60 females; age average: 21.1 ± 2.9 years) who attended Dicle University, Faculty of Dentistry, Clinic of Orthodontics for Orthodontic Treatment, participated in the present study. The subject participation in this study was retrospectively selected among patients that indicate skeletal Cl I, II, and III relationship. The radiographic data included lateral cephalometric radiographs. The criteria for selection of patients' radiographs had to be of high quality and sharpness, and all radiographs had to be taken by the same apparatus and same technician, with patients in natural head posture. Natural head posture was determined by using a fluid level method as described by Showfety et al. [27].

The patients were divided into three groups (Cl I, II, and III), *n* = 40 (*n* = 20 female, *n* = 20 male) subjects each, according to a widely used indicator of skeletal anterior-posterior discrepancies known as the ANB angle. The ANB angle refers to the angle opened between the A-point, the nasion, and the B-point on a cephalograph. This value was used by Steiner [28–30] as means to group participants according to skeletal class: Class I, ANB = 2–4°; Class II, ANB > 4°; and Class III, ANB < 2°. In addition, each group was divided into subgroups according to sex (20 subjects each). All participants enrolled in this study showed normal vertical skeletal patterns (SNGoMe = 28–36°), had not previously undergone orthodontic treatment or orthognathic surgery, showed no visual, hearing, breathing, or swallowing disorders, and had no respiratory disturbance or any other trauma affecting the craniofacial region.

**Figure 1:**
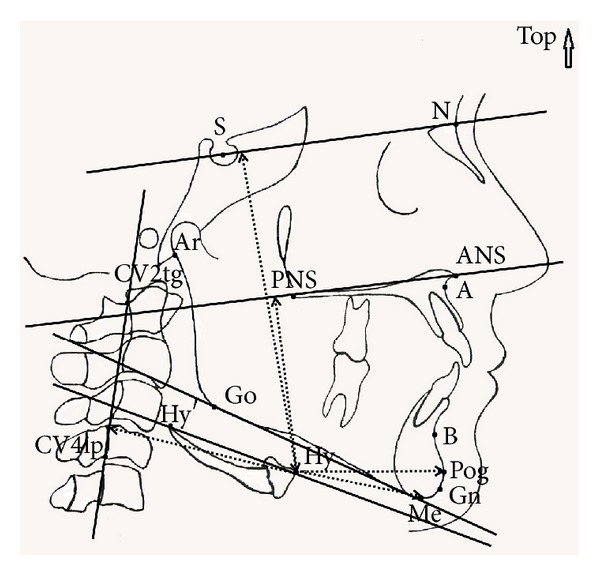
Angular and linear measurements used.

**Figure 2:**
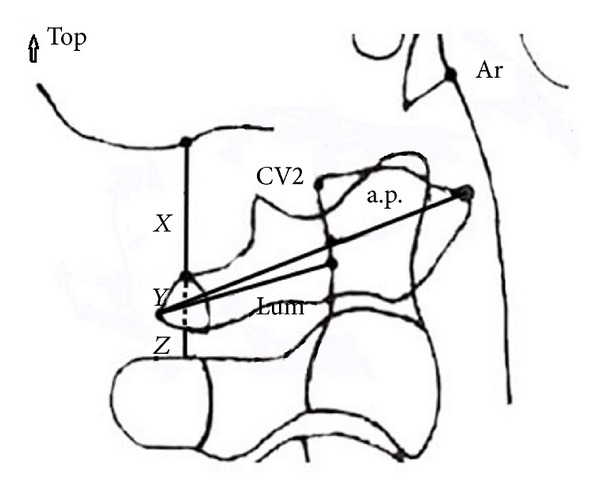
Linear measurements on the first cervical vertebrae.

All cephalometric tracings were performed by the same person (SGA) using 0.03 mm matte acetate paper with a 3H pencil. Eleven linear measurements were performed as described by Ceylan [31]. Based on the landmarks and lines defined in Tables 1 and 2, respectively, a total of 14 angular and linear measurements were assessed as described in Table 3. The cephalometric landmarks and measurements were shown in Figures 1 and 2.

Two weeks after the first measurements, 30 radiographs were selected at random and remeasured, and a correlation analysis was performed using data from the first and second measurements. Differences observed between the first and second measurements were not significant (*P* > 0.05).

All statistical analyses were performed using the Statistical Package for Social Sciences version 10.0 (SPSS, Chicago, Illinois, USA). Gender differences among groups were analyzed using paired-sample* t*-tests. Mean differences among groups were examined by analysis of variance (ANOVA) for repeated measures with a* post hoc* least significant difference (LSD) test for multiple comparisons. The level of significance for all analyses was set at *P* < 0.05.

## 3. Results

Intragroup (male versus female participants) and intergroup comparisons of differences between different skeletal patterns are shown in Tables 4, 5, and 6. Intragroup comparison of Class I male and female participants revealed statistically significant differences in the Hy-NSL (*P* < 0.001), Hy-PD (*P* < 0.001), Hy-CVT (*P* < 0.001), *X* (*P* = 0.004), *Y* (*P* = 0.012), Lum (*P* = 0.013), and a-p (*P* = 0.001) measurements. Although the hyoid bone was positioned more inferiorly and anteriorly in males than in females with Class I skeletal patterns, the dimensional measurements for C1 revealed that this vertebra was larger and positioned lower in males. In the intragroup comparison of Class II participants, significant differences were observed for Hy-NSL (*P* = 0.006), Hy-NL (*P* = 0.050), and Hy-CVT (*P* = 0.001). The measurements Hy-NSL (*P* < 0.001), Hy-NL (*P* = 0.003), Hy-CVT (*P* < 0.001), Lum (*P* = 0.027), and a-p (*P* = 0.29) also showed statistically significant differences in the intragroup comparison for Class III participants. All measurements were greater in magnitude for male versus female participants. The hyoid was positioned more inferiorly and anteriorly in males with Class II and Class III skeletal patterns. In addition, C1 was larger in the anterior-posterior direction in males versus females with Class III skeletal patterns.

Intergroup comparison showed statistically differences in the SNA, ANB, Hy-CVT, *X*, and *Z* measurements in male participants. In addition, SNA showed statistically significant differences between Class II and Class III participants (*P* = 0.006) and between Class I and Class III participants (*P* = 0.003). ANB also revealed statistically significant differences among all groups (*P* < 0.001). Hy-CVT showed statistically significant differences between Class I and Class II participants (*P* = 0.019). The *X* and *Z* measurements were significantly different between Class I versus Class III (*P* = 0.010) and Class II versus Class III (*P* = 0.004) participants, respectively. The hyoid was positioned more posteriorly in participants with Class II skeletal patterns compared to those with Class I skeletal patterns. In addition, the posterior part of C1 was positioned more inferiorly among participants with Class I versus Class III skeletal patterns. C1 was also closer to C2 among participants of Class II versus Class III skeletal patterns. In contrast, the intergroup comparison among female participants revealed a significant difference in only the ANB measurement among the groups.

## 4. Discussion

The position of the hyoid bone is of great clinical interest because it plays an important role in maintaining the dimensions of the upper airway and an upright natural head posture [26, 32, 33]. The first cervical vertebra, also known as the atlas, represents the transition between the skull and the axial skeleton and it is reported that the dimensions of this vertebra affect aspects of both the facial skeleton and the cervical column [34]. Numerous previous studies have shown that orthodontic treatments impacting mandibular position can also alter the position of the hyoid, given that it is attached to the mandible via the geniohyoid, anterior digastric, and mylohyoid muscles [35–37].

Graber [18] evaluated the position of the hyoid in 30 children (16 males and 14 females, mean age of 6 years) after orthopedic treatment for mandibular prognathism. Graber observed that the hyoid position had shifted posteriorly and inferiorly at 3 years after treatment. Sürücü et al. [38] examined 10 individuals with skeletal Class II division I malocclusions and found that, after the activator was applied and the bite was opened, the tongue shifted posteriorly and caused constriction of the upper airway. To compensate for this constriction, patients would need to alter their normal upright head posture by extending the head/jaw. The authors reported that, as a result of these changes, the mandible moved into a more anterior position, while the hyoid shifted anteriorly depending on the degree of head extension. It has been reported that the hyoid moves posteriorly and inferiorly in mandibular setback surgeries [39, 40]. This subsequently results in the base of the tongue shifting posteriorly and inferiorly, resulting in constriction of the upper airway and forcing the patient to further extend the head position to lengthen the upper airway. Posterior movement of the mandible can cause relaxation of the suprahyoid musculature, which may instigate balance disorders in the head and neck muscles and oropharyngeal complex. It has been postulated that if this relaxation continues for an extended period, it can alter the position of the hyoid as well as the length of the suprahyoid muscle, causing skeletal relapse [41].

Cephalometric radiography, one of the most important tools in orthodontics both clinically and in research, permits the accurate evaluation of the dental, skeletal, and soft tissue relationships of the craniofacial complex before the initiation of treatment and during growth [42–44].

In light of this information, the aim of this study was to document the position of the hyoid bone and cervical atlas morphology on the cephalometric radiographs of participants with different sagittal skeletal patterns, with the goal of identifying cephalometric norms. To achieve this, participants were classified according to horizontal discrepancies observed in the ANB angle, a parameter that is widely used in the evaluation of skeletal anterior-posterior relationships [45–47]. Previous studies have shown that natural head posture is related to respiratory [48] and visual function [49]. Therefore, to determine natural head posture in a precise manner, only individuals without visual, hearing, or respiratory disturbances were included in this study.

Our results from the intragroup comparison of Class I and Class II participants revealed that several measurements were significantly larger in males than in females. These included Hy-NSL (*P* < 0.001), Hy-PD (*P* = 0.001), and Hy-CVT (*P* < 0.001). In addition, the hyoid was positioned more anteriorly and inferiorly in males than in females. Şahin Sağlam and Uydas [50] investigated variation in hyoid position and head posture between genders (38 females and 38 males) using lateral cephalometric films obtained using a natural head position. All participants had a Class I skeletal pattern and ideal dental occlusion. The authors found that natural head position did not differ between genders, but the hyoid bone was positioned more superiorly and posteriorly in females compared to males. Ceylan [51] examined 90 adolescents, including 45 boys and 45 girls aged 13–15 years and analyzed the relationship of the ANB angle and natural head position to the position of the hyoid bone. Natural head position was not significantly affected by gender, but the hyoid bone was positioned significantly more superiorly and posteriorly in females than in males. These results are consistent with the data reported here.

In the intragroup comparison of Class I participants, significant differences were observed with respect to the *X*, *Y*, Lum, and a-p parameters, all of which were smaller in females. The data also revealed a greater superior-posterior area of C1, shorter distance between C1 and C2, shorter anterior-posterior dimensions, and shorter distance between the anterior border of the C1 dorsal arch and the posterior border of the C2 odontoid process also in females. These results suggest that the male body is more developed than the female body.

In the intragroup comparison of Class III participants, it was found that the hyoid bone was positioned more anteriorly and inferiorly in males than in females. Cervical and craniocervical posture may be one factor explaining the significant differences observed between males and females who have Class III malocclusions [24]. Females who have Class III malocclusions show a much more “normal” position of the hyoid bone as compared to males with Class III malocclusions. This may be due to heightened aesthetic awareness of a prognathic mandible in girls, which leads them to assume, quite early in life, a modified head posture allowing them to minimize the appearance of a prominent chin.

Intergroup comparisons of differences between genders showed statistically significant differences in ANB measurements in all groups. These results are unsurprising given that participants with normal vertical patterns and different ANB values were included in this study. In the intergroup comparison of males, the Hy-CVT distance was found to be greater in Class I than in Class II participants. This indicates pronounced anterior positioning of the hyoid bone in Class I males. On the other hand, Ceylan [31] evaluated the hyoid bone position in different skeletal patterns of adolescents aged 13–15 years and reported that the hyoid bone was positioned more anteriorly with an increase in the ANB angle. This is in direct contrast to our study, which may be the result of examining different age groups (adolescents versus young adults) and the inclusion of participants with normal vertical skeletal patterns in our work. Dincer et al. [52] investigated the position of the hyoid bone with respect to its relationship with the dentofacial system in 45 participants with Class I, Class II division I, and Class III malocclusions. It was ascertained that the hyoid was positioned more posteriorly among participants with a Class II division I skeletal pattern compared to a Class I pattern and more anteriorly among participants with a Class III skeletal pattern compared to a Class I pattern. In addition, the greater *X* values among Class I versus Class III participants indicate an overall lower position of C1 in the Class I group. Class III participants had higher *Z* values compared to the Class II group; accordingly, the distance between the posterior part of C1 and C2 is greater in Class III versus Class II participants.

Regarding skeletal patterns in female participants, no difference was observed for parameters relating to the hyoid or C1, indicating no morphological difference in these structures among females. Consistent with our findings, Adamidis and Spyropoulos [24] found no statistically significant differences in the position or orientation of the hyoid among females exhibiting Class I malocclusions versus those with Class III malocclusions.

## 5. Conclusions 

This study revealed that the hyoid bone is positioned more inferiorly and anteriorly and is more prominent in males than in females, regardless of variation in the axial skeleton. C1 was also larger along the anterior-posterior axis in males exhibiting a Class I skeletal pattern compared to females in the same class. However, no significant difference in hyoid position or C1 morphology was observed in relation to the ANB in females. In the sagittal plane, the hyoid bone was similar in males with a Class I or Class III skeletal pattern but was positioned more posteriorly, based on mandibular bone development, in males with Class I skeletal patterns. In addition, the vertical position of C1 varied depending on the sagittal skeletal pattern among male participants.

It is especially important among males that the hyoid bone remains in the same position after orthodontic and orthognathic treatment so that the balance of the soft tissues is not altered, thus, reducing the chance of relapse. The results of this study are therefore highly useful for clinicians who must assess the stability of these structures in patients who are candidates for orthognathic or orthopedic surgery, which may help to prevent the obstruction of the upper airway. In addition, this study helps to identify cephalometric norms that exist among males and females who have different skeletal sagittal anomalies.

## Figures and Tables

**Table 1:** Description of landmarks.

Landmark	Definition
Nasion (N)	Most anterior point of the frontonasal suture
Sella (S)	Center of the sella turcica
Articulare (Ar)	Point of intersection between the posterior border of the mandibular ramus and the inferior border of the posterior cranial base
Spina nasalis anterior (ANS)	
Spina nasalis posterior (PNS)	
Pogonion (Pog)	Most anterior point on the mandibular symphysis
Gnathion (Gn)	Most anterior-inferior point on the mandibular symphysis
Menton (Me)	Most inferior point on the mandibular symphysis
Hyoid (Hy)	Most superior-anterior point on the body of the hyoid bone
Hyoid′ (Hy′)	Most superior-posterior point on the grater horn of hyoid bone
CV4ip	Most poster-inferior point on the corpus of the fourth cervical vertebra
CV2tg	Most superior-posterior point on the corpus of the fourth cervical vertebra

**Table 2:** Description of lines.

Line	Definition
CVT (the upper part of the cervical spine)	Line through CV2tg and CV4ip in the upper part of the cervical spine
NSL (nasion-sella line)	The nasion-sella line through points N and S
NL (nasal line)	The line through ANS and PNS
ML (mandibular line)	The mandibular line, tangential to the lower border of the mandible through Gn
Hy-Hy′ (hyoid line)	The hyoid line passing through Hy and Hy′

**Table 3:** Description of linear measurements.

Landmark	Definition
Hy-NSL	Linear distance from H along a perpendicular to NSL
Hy-NL	Linear distance from H along a perpendicular to the NL
Hy-Hy′/ML	Linear distance from hyoid line to mandibular line
Hy-CVT	Linear distance from H along a perpendicular to CVT
Hy-Pog	The linear distance between H and pogonion
Hy-Me	The linear distance between H and menton
*X*	Distance between inferior border of the occipital bone and dorsal arch of first cervical vertebrae
*Y*	Distance between most superior and inferior points of first cervical vertebrae's dorsal arch
*Z*	Distance between most inferior point of first cervical vertebrae's dorsal arch and most superior point of second cervical vertebrae's (axis) spinal process
Lum	Distance between anterior border of the first cervical vertebrae's dorsal arch and posterior border of the second cervical vertebrae's odontoid process
a-p	Distance between first cervical vertebrae's most frontal point of tuberculum anterior and most posterior point of dorsal arch

**Table 4:** Intragroup comparison of male and female participants.

Measurements	Class I						Class II						Class III					
	Female (*n* = 20)		Male (*n* = 20)		(*P* value)		Female (*n* = 20)		Male (*n* = 20)		(*P* value)		Female (*n* = 20)		Male (*n* = 20)		(*P* value)	
	Mean	SD	Mean	SD			Mean	SD	Mean	SD			Mean	SD	Mean	SD		
SNA (°)	80,80	4,46	83,60	5,03	0,268	NS	83,30	5,39	83,37	3,81	0,858	N.S	79,00	4,85	76,77	4,35	0,434	N.S
SNB (°)	77,90	4,48	80,40	4,85	0,053	N.S	76,60	5,60	76,43	3,35	0,858	N.S	80,80	3,91	78,88	4,16	0,510	N.S
ANB (°)	2,90	1,28	3,20	1,01	0,240	N.S	6,70	0,67	6,93	1,04	0,776	N.S	−1,80	2,04	−2,11	0,78	0,127	N.S
Hy Hy′ MD (°)	7,70	5,37	5,40	2,91	0,424	N.S	8,40	3,89	11,75	9,54	0,624	N.S	8,40	5,37	11,77	9,93	0,624	N.S
Hy-NSL (mm)	103,70	4,27	120,60	7,91	0,000	∗∗∗	105,60	5,54	118,87	10,52	0,006	∗∗	106,00	5,22	119,66	7,56	0,001	∗∗
Hy NL (mm)	59,50	4,67	71,10	6,67	0,001	∗∗	63,50	6,67	71,62	9,30	0,050	∗	62,10	5,08	71,77	6,24	0,003	∗∗
Hy-CVT (mm)	49,40	2,79	59,30	2,66	0,000	∗∗∗	50,20	2,78	56,12	1,64	0,001	∗∗	49,90	2,68	58,66	3,27	0,000	∗∗∗
Hy Pog(mm)	54,90	8,18	51,10	3,60	0,196	N.S	51,60	3,56	50,25	6,47	0,964	N.S	51,90	5,40	49,77	5,91	0,566	N.S
Hy Me (mm)	49,10	7,24	43,60	3,68	0,063	N.S	44,40	3,94	42,75	6,25	0,562	N.S	45,20	4,31	43,66	5,93	0,623	N.S
*X* (mm)	8,00	1,63	11,70	2,62	0,004	∗∗	8,60	1,95	9,37	2,87	0,743	N.S	9,10	2,13	8,22	2,63	0,677	N.S
*Y* (mm)	8,30	1,25	10,40	1,71	0,012	∗	8,70	1,49	9,75	2,37	0,470	N.S	8,50	1,43	9,66	1,22	0,077	N.S
*Z* (mm)	7,00	2,21	7,30	2,26	0,908	N.S	6,50	2,41	5,87	2,41	0,369	N.S	7,80	1,47	9,11	1,61	0,086	N.S
LUM (mm)	27,90	2,72	31,20	2,29	0,013	∗	28,4	2,22	29,87	2,85	0,227	N.S	29,00	3,09	34,00	6,83	0,027	∗
a-p (mm)	46,90	1,85	51,90	2,46	0,001	∗∗	48,50	2,01	50,75	3,99	0,192	N.S	47,40	2,41	49,88	6,64	0,029	∗

*P < 0.05, **P < 0.01, ***P < 0.001, N.S.: nonsignificant, SD: standard deviation, and *n*: number of patients.

**Table 5:** Intergroup comparison of mean differences among males with Class I, II, and III skeletal patterns.

Measurements	Class I (*n* = 20)		Class II (*n* = 20)		Class III (*n* = 20)		ANOVA	Multiple comparison LSD test (*P* value)		
	Mean	SD	Mean	SD	Mean	SD		CI-CII	CII-CIII	CI-CIII
SNA (°)	83,60	5,03	83,38	3,81	76,78	4,35	∗∗	0,917	0,006	0,003
SNB (°)	80,40	4,85	76,44	3,35	78,89	4,16	N.S	0,060	0,245	0,445
ANB (°)	3,20	1,31	6,94	1,14	−2,11	0,78	∗∗∗	0,000	0,000	0,000
Hy Hy′ MD (°)	5,40	2,91	11,75	9,54	11,78	9,93	N.S	0,104	0,994	0,092
Hy NSL (mm)	120,60	7,91	118,88	10,52	119,67	7,56	N.S	0,678	0,852	0,816
Hy NL (mm)	71,10	6,67	71,62	9,30	71,78	6,24	N.S	0,883	0,967	0,844
Hy-CVT (mm)	59,30	2,66	56,12	1,64	59,67	2,27	∗	0,019	0,060	0,608
Hy Pog (mm)	51,10	3,60	50,25	6,47	49,78	5,91	N.S	0,741	0,858	0,596
Hy Me (mm)	43,60	3,68	42,75	6,25	43,67	5,93	N.S	0,739	0,726	0,978
*X* (mm)	11,70	2,62	9,38	2,87	8,22	2,63	∗	0,082	0,389	0,010
*Y* (mm)	10,40	1,71	9,75	2,37	9,67	1,22	N.S	0,454	0,925	0,384
*Z* (mm)	7,30	2,26	5,88	2,41	9,11	1,61	∗	0,169	0,004	0,075
LUM (mm)	31,20	2,30	29,88	2,85	34,00	6,83	N.S	0,537	0,069	0,185
a-p (mm)	51,90	2,47	50,75	3,99	49,89	6,64	N.S	0,607	0,707	0,356

*P < 0.05, ∗∗*P* < 0.01, ∗∗∗*P* < 0.001, N.S.: statistically not significant, SD: standard deviation, and *n*: number of patients.

**Table 6:** Intergroup comparison of mean differences among females with Class I, II, and III skeletal patterns.

Measurements	Class I (*n* = 20)		Class II (*n* = 20)		Class III (*n* = 20)		ANOVA	Multiple comparison LSD test (*P* value)		
	Mean	SD	Mean	SD	Mean	SD		CI-CII	CII-CIII	CI-CIII
SNA (°)	80,80	4,46	83,30	5,39	79,00	4,85	N.S	0,266	0,061	0,421
SNB (°)	77,90	4,48	76,60	5,6	80,80	3,91	N.S	0,895	0,072	0,094
ANB (°)	2,90	1,28	6,70	0,67	−1,80	1,04	∗∗∗	0,000	0,000	0,000
Hy Hy′ MD (°)	7,70	5,37	8,40	3,89	8,40	5,37	N.S	0,753	1.000	0,753
Hy NSL (mm)	103,70	4,27	105,60	5,54	106,00	5,22	N.S	0,407	0,861	0,317
Hy NL (mm)	59,50	4,67	63,50	6,67	62,10	5,08	N.S	0,118	0,577	0,304
Hy-CVT (mm)	49,40	2,79	50,20	2,78	49,90	2,68	N.S	0,522	0,809	0,688
Hy Pog (mm)	54,90	8,18	51,60	3,56	51,90	5,40	N.S	0,231	0,912	0,275
Hy Me (mm)	49,10	7,24	44,40	3,95	45,20	4,31	N.S	0,061	0,742	0,117
*X* (mm)	8,00	1,63	8,60	1,95	9,10	2,13	N.S	0,490	0,565	0,211
*Y* (mm)	8,30	1,25	8,70	1,49	8,50	1,43	N.S	0,527	0,751	0,751
*Z* (mm)	7,00	2,21	6,5	2,41	7,8	1,47	N.S	0,594	0,172	0,396
LUM (mm)	27,90	2,72	28,4	2,22	29,00	3,09	N.S	0,682	0,624	0,371
a-p (mm)	46,90	1,85	48,50	2,01	47,40	2,41	N.S	0,101	0,253	0,600

***P < 0.001, N.S.: statistically not significant, SD: standard deviation, and *n*: number of patients.
